# A circumpolar review of the breeding distribution and habitat use of the snow petrel (*Pagodroma nivea*), the world’s most southerly breeding vertebrate

**DOI:** 10.1007/s00300-024-03336-8

**Published:** 2024-12-18

**Authors:** Josie Francis, Ewan Wakefield, Stewart S. R. Jamieson, Richard A. Phillips, Dominic A. Hodgson, Colin Southwell, Louise Emmerson, Peter Fretwell, Michael J. Bentley, Erin L. McClymont

**Affiliations:** 1 Department of Geography, Durham University, South Road, Durham, DH1 3LE UK; 2 British Antarctic Survey, High Cross Madingley Road, Cambridge, CB3 0ET UK; 3 Australian Antarctic Division, Kingston, TAS Australia

**Keywords:** Breeding distribution, Climate, Habitat, Antarctica, Sea-ice conditions, Snow petrel

## Abstract

Knowledge of the spatial distribution of many polar seabird species is incomplete due to the remoteness of their breeding locations. Here, we compiled a new database of published and unpublished records of all known snow petrel *Pagodroma nivea* breeding sites. We quantified local environmental conditions at sites by appending indices of climate and substrate, and regional-scale conditions by appending 30 year mean (1992–2021) sea-ice conditions within accessible foraging areas. Breeding snow petrels are reported at 456 sites across Antarctica and subantarctic islands. Although many counts are old or have large margins of error, population estimates available for 222 known sites totalled a minimum of ~ 77400 breeding pairs. However with so many missing data, the true breeding population will be much higher. Most sites are close to the coast (median = 1.15 km) and research stations (median = 26 km). Median distance to the November sea-ice edge (breeding season sea-ice maximum) is 430 km. Locally, most nests occur in cavities in high-grade metamorphic rocks. Minimum air temperatures occur at inland sites, and maxima at their northern breeding limit. Breeding location and cavity selection is likely determined by availability of suitable breeding substrate within sustainable distance of suitable foraging habitat. Within this range, nest sites may then be selected based on local conditions such as cavity size and aspect. Our database will allow formal analyses of habitat selection and provides a baseline against which to monitor future snow petrel distribution changes in response to climate change.

## Introduction

Globally, seabirds are one of the most threatened marine taxonomic groups (Sydeman et al. 2012; Dias et al. 2019). However, knowledge of their spatial distribution and population sizes is incomplete, particularly in remote, often inaccessible locations such as Antarctica (Rodríguez et al. 2019). Satellite remote sensing in Antarctica has enabled the discovery and estimation of population sizes for colonies of several surface-nesting species: Adélie penguins *Pygoscelis adeliae*, emperor penguins *Aptenodytes forsteri*, chinstrap penguins *P. antarcticus* and Antarctic petrels *Thalassoica antarctica* (Schwaller et al. 1989, 2013, 2018; Fretwell and Trathan 2009; Fretwell et al. 2012, 2015; Lynch and LaRue 2014; Román et al. 2022). However, knowledge of the circumpolar distributions of smaller cavity-nesting or burrowing seabirds remains largely reliant on direct observations (Southwell et al. 2011; Barbraud et al. 2018). In the Southern Ocean, seabirds are often considered to be useful indicators of ecosystem health, and species distribution data are critical to conservation and management (González-Zevallos et al. 2013; Pande and Sivakumar 2022). Our focus here is on defining the breeding distribution of the most southerly breeding vertebrate, the snow petrel *Pagodroma nivea*, which was last reviewed almost three decades ago (Croxall et al. 1995). Since then, scientific research has intensified on the continent and several targeted surveys have been undertaken (Barbraud et al. 1999; Convey et al. 1999; Olivier et al. 2004; Pande et al. 2020). Given the importance of baseline distribution data for seabird monitoring, and because observations have continued to be made in the intervening period, it is now timely to provide an updated review of the known circumpolar snow petrel breeding distribution.

Snow petrels are a high-trophic-level seabird endemic to Antarctica with a northern breeding limit in South Georgia (Croxall et al. 1995). They have one of the highest affinities for pack ice of all Antarctic seabirds, feeding predominantly on fish, krill, and squid in proportions that vary dependent on foraging location (Ainley and Jacobs 1981; Ainley et al. 1984; Ridoux and Offredo 1989). When foraging at sea, snow petrels are largely confined to the Marginal Ice Zone [MIZ] and in particular, intermediate sea-ice concentrations of 12.5–50% (Zink 1981; Ainley et al. 1984, 1998). Like all seabirds, their foraging ranges during the breeding season are limited by the central-place constraint (Delord et al. 2016). Variability in sea-ice conditions within their foraging areas, both prior to and during the breeding season, affects annual adult survival, colony size, and breeding phenology (Barbraud et al. 2000; Barbraud and Weimerskirch 2001; Jenouvrier et al. 2005; Sauser et al. 2021b).

Snow petrels are cavity nesters, requiring ice-free areas for breeding (Walton 1984). The lithology and geomorphology at breeding sites is thus important in determining cavity presence. Nesting cavities occur in cliff faces, on scree slopes, and under boulders on flat and sloping ground. Characteristics such as slope, aspect, number of entrances, and nest bowl slope vary within and among breeding sites. However, nests with single, narrow entrances are used more frequently, and hatching success and chick survival are greatest when nest bowls are flat (Jouventin and Bried 2001; Einoder et al. 2014). Local meteorological conditions can affect access to nests or cause breeding failure (Sydeman et al. 2012), and it has been suggested that the combination of nest aspect and local wind direction is critical in ensuring that cavities remain snow-free for breeding (Olivier and Wotherspoon 2006). However, the relationship is not consistent; in the Windmill Islands, most snow petrel nesting cavities are oriented towards strong prevailing winds (Cowan 1981), whereas in the Bunger Hills and Dronning Maud Land they are typically oriented for protection from prevailing katabatic winds (Wand and Hermichen 2005). Variability in local climatic conditions during the breeding season, including timing, intensity and duration of precipitation, wind speed, direction and duration, and local air temperatures, affect snow petrel breeding phenology and demography (Chastel et al. 1993; Sauser et al. 2021a, b). Baseline knowledge of conditions in the foraging and breeding habitats of snow petrels is therefore required for predicting how populations are likely to respond to future environmental changes at sea and on land.

In the only comprehensive review to date, snow petrels were confirmed as breeding at 195 sites across Antarctica and subantarctic islands, and suspected to breed at another 103 localities, from which the authors concluded that the minimum known total breeding population was 63000 pairs (Croxall et al. 1995). Typically, a large proportion (> 50%) of petrel populations is represented by non-breeders (juveniles, immatures, and non-breeding adults) (Phillips et al. 2017; Carneiro et al. 2020), and based on regional at-sea counts (Ainley et al. 1984; Cooper and Woehler 1994), a total population size of several million birds was estimated (Croxall et al. 1995). However, regional breeding populations are often much smaller than at-sea densities would suggest. For example, 1.97 million snow petrels were estimated in the Ross Sea area from counts at sea, but in this region only 14 breeding sites totalling ~ 5300 breeding pairs were recorded, suggesting many breeding sites may remain undetected (Ainley et al. 1984; Croxall et al. 1995).

The primary aim of this study was to (1) review the known global breeding distribution and habitat use of snow petrels. This includes exploring previously unquantified relationships between lithology and cavity availability, and between foraging habitat use and the distribution of known sites. To do so, we first collated records of breeding locations, including population estimates when available. Due to a paucity of reliable count data at most sites and a lack of longitudinal count data at all but a handful of sites, we did not aim to produce a comprehensive population estimate nor examine population change over time. Our subsidiary aims were to (2) characterise the local environmental conditions at breeding sites (specifically lithology and climate variables including temperature, precipitation and wind speed) and distance from the coast, and (3) characterise regional sea-ice conditions within areas accessible from each site. By doing so, we describe both the breeding habitat use and distribution of snow petrels throughout their circumpolar range. All data are presented in an accompanying open access database (Francis et al. 2024), which we hope will facilitate ongoing research and conservation.

## Methods

### Database compilation

To determine the known breeding distribution, an intensive search of the published literature and archived field reports was conducted, and all identified breeding sites were incorporated into a database with the following information: Site name and decimal coordinates; site aspect, elevation and local lithology; and when survey data were available, nest density. Snow petrel nest densities range from highly dispersed (0.3 nests per ha) to relatively dense aggregations (24.1 nests per ha) (Olivier et al. 2004; Olivier and Wotherspoon 2008), and uncalculated densities may be higher. However, even the maximum densities do not reach the high densities of colonies of the Antarctic petrel, which is closely related and also breeds in the region (Mehlum et al. 1988; Schwaller et al. 2018). Therefore, it is difficult to define the spatial extent of a snow petrel colony, and to avoid ambiguity, we use the term ‘breeding site’ instead of ‘colony’, where a breeding site is defined as a locality with individual coordinates where breeding is likely or confirmed (based on observations).

Archived field reports, field notebooks, and maps from 1945 onwards at the British Antarctic Survey were searched to extract relevant spatial data, including from locations provided by Croxall et al. (1995). We also contacted seabird biologists with field knowledge of the Antarctic region and included their unpublished observations.

In all cases, we included the most recent quantitative data (e.g., coordinates, estimates of population size) for a specific breeding site in the database. Additional fields included breeding site identification [IDs] and Antarctic Conservation Biogeographic Region / Benthic Biogeographic Region (Terauds and Lee 2016; Convey et al. 2014). For breeding sites between 30°E and 150°E, fields of ‘Spatial sub-group’ and ‘Site_ID(s)’ were added to conform with the spatial reference system of Southwell et al. (2021). At each locality, we distinguished whether breeding was confirmed or unconfirmed. For breeding to be confirmed, observations of active nests and the presence of eggs or chicks had to be reported. Otherwise, where nests were suspected but not found (e.g., Moss Island (González-Zevallos et al. 2013)), or breeding was either not mentioned or reported to be likely or possible (e.g., Stinear Peninsula (Pande et al. 2020)), breeding was recorded as unconfirmed. Sites that were checked but there was no evidence of breeding (i.e., during dedicated surveys) were recorded as absences.

### Local environmental conditions

To describe breeding habitat use at the local scale, climate and lithology at the breeding sites were quantified.

Climate reanalysis data for the period 1992–2021 were obtained from the ERA5-Land monthly averaged dataset, Copernicus (Muñoz Sabater 2019), including: 2m surface temperature, total precipitation, and 10m wind speed and direction. Seasonal 30-year averages and summary statistics for each variable were then calculated for each breeding site. The breeding season was defined as November-March, which covers the period between arrival of adults and chick fledging (Olivier et al. 2005).

Lithological data were extracted from the SCAR GeoMAP shapefile, comprising the known geology of all Antarctic bedrock and surficial deposits (Cox et al. 2023a). Breeding site lithologies were subsequently grouped into 8 categories for analysis, according to the simple lithological description in Cox et al. (2023b). In order to determine if habitat use reflected availability, the relative frequency distribution of lithology at breeding sites was compared to that within all exposed rock polygons.

### Regional sea-ice conditions

To characterise the foraging habitat available to snow petrels at each breeding site, we assumed a mean and maximum summer foraging range of 700 km and 1500 km, respectively (Delord et al. 2016; authors’ unpublished GPS tracking data).

Passive microwave sea-ice data for the years 1992–2021 were acquired from the National Snow and Ice Data Centre (NSIDC). Sea-ice conditions were based on 30-year averages in November and February—chosen as the points in the breeding season when sea-ice extent [SIE] is at its maximum and minimum, respectively. We focused on the low sea-ice concentration [SIC] MIZ most commonly used by breeding snow petrels for foraging. For November and February, we calculated the contours at the outer ice edge with 15% SIC (Olivier et al. 2005), and 50% SIC, because these correspond to the two habitats where the highest at-sea densities of snow petrels are recorded (Zink 1981). We also generated the associated rasters of SIC. We calculated the distance from breeding sites to the 15 and 50% SIC contours for these months in 1992–2021, then calculated the average over the 30 years. Finally, we estimated the foraging area within the mean and maximum foraging ranges of each breeding site, using buffers of 700 and 1500 km from breeding sites, by counting the number of pixels between 15–50% SIC for the relevant months between 1992 and 2021, and transforming to an area by multiplying by the area of a single pixel (625 km^2^). For all sea-ice metrics, results were plotted by frequency, and summarised by calculating the median, interquartile range [IQR], and range. All analyses were carried out in QGIS version 3.26.3 (QGIS Development Team 2023) and R version 4.2.2 (R Core Team 2022).

## Results

### Spatial distribution and size of breeding sites

**Fig. 1:**
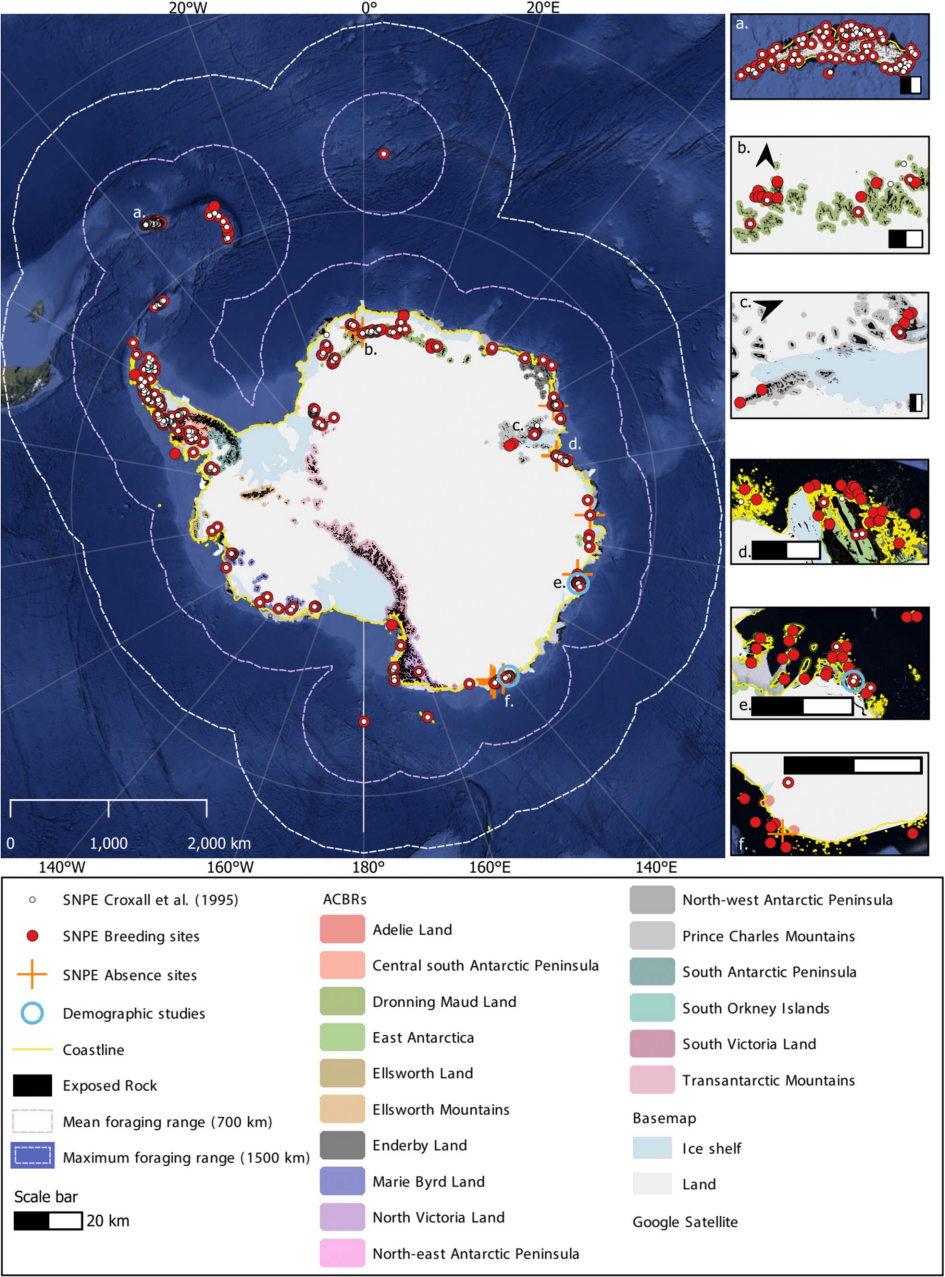
The updated breeding distribution of snow petrels [SNPE]. Each dot represents one breeding site. The breeding sites from Croxall et al. (1995) are shown in white, and the updated breeding distribution in red (456 breeding sites). Regional insets for **a** South Georgia, **b** Dronning Maud Land, **c** Inland Prince Charles Mountains, **d** and **e** East Antarctica, and **f** Adélie Land, show new breeding sites. Known absences shown by orange crosses. Coastline is combined data from the SCAR Antarctic Digital Database (accessed 2023, Gerrish et al. 2022), and Thematic Mapping World Borders (accessed 2023). Exposed rock is sourced from Cox et al. (2023a); Antarctic Conservation Biogeographic Regions [ACBRs] are sourced from Terauds and Lee (2016); basemap from NPI/Quantarctica, and underlying imagery is Google Satellite. Map projection is Antarctic Polar Stereographic

Our database represents a considerable expansion in knowledge of the global breeding distribution of snow petrels (Fig. 1) since Croxall et al. (1995). We list 456 confirmed and suspected (snow petrels observed but breeding unconfirmed) breeding sites. Of these, 158 are newly identified, principally in Dronning Maud Land (28 new sites), the Prince Charles Mountains (11 new inland, 43 new coastal sites), and Adélie Land (19 new sites). Additionally, surveys in localities such as the Larsemann Hills (Pande et al. 2020) have enabled separation of a single breeding site in Croxall et al. (1995) into multiple sites in our database. Of the 456 known sites, breeding was confirmed at 267 (59%), and unconfirmed (but suspected) at the remaining 189. Most breeding sites (74%) are located around the Antarctic continent, and 120 (26%) on islands (Bouvet Island, Balleny Islands, South Orkney Islands, South Sandwich Islands, South Georgia). However, when considering the total population estimate (combined counts over time), just 51% of known breeding pairs are (or were) on the continent—noting that population estimates are only available for 55% of continental breeding sites, and that the estimate of 20000 breeding pairs on Laurie Island (South Orkney Islands) constitutes a large proportion of the known breeding population.

**Fig. 2:**
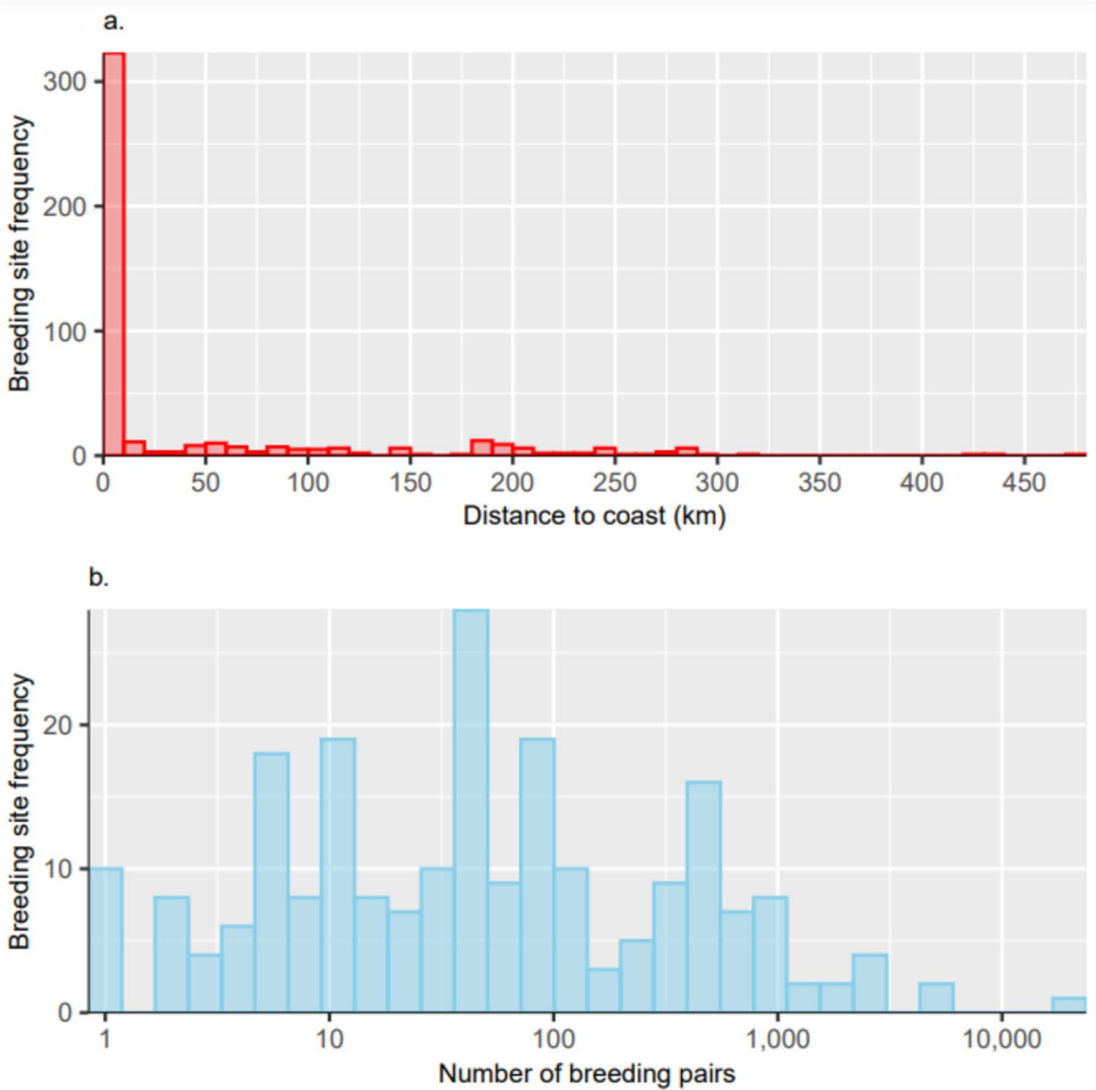
**a** Frequency distribution of distance from each breeding site to the coast, and **b** frequency distribution of the number of breeding pairs at each breeding site on a logarithmic scale

The median distance of breeding sites from the coastline (based on Gerrish et al. 2023) was 1.15 km (IQR = 0.23 to 42.75 km, range = 0.00 to 471.27 km, *n* = 456). Prior to 1995, the furthest known inland breeding sites were in the Tottanfjella, Dronning Maud Land, over 300 km from the coast (Bowra et al. 1966). Although most known breeding sites are very close to the coast (Fig. 2a), a small breeding site exists 440 km inland at Greenall Glacier, Mawson Escarpment, and an unconfirmed breeding site at Rimington Bluff (470 km inland) in the inland Prince Charles Mountain (Goldsworthy and Thomson 2000). The site at Greenall Glacier increases the distance inland at which snow petrels are known to breed by 140 km.

The number of breeding pairs is extremely variable among sites (median = 50, IQR = 10 to 171, range = 1 to 20000, *n* = 222; Fig. 2b). At some, single breeding pairs were recorded (e.g., Orvinfjella region, Dronning Maud Land; Dragons Teeth Cliffs, Prince Charles Mountains; Mount Haskel, north-west Antarctic Peninsula). In contrast, 4,575 breeding pairs were estimated on Browning Peninsula, South Windmill Islands (Olivier et al. 2004), and 20000 breeding pairs on Laurie Island, South Orkney Islands (Clarke 1906; Croxall et al. 1995). However, the number of breeding pairs is only known (counts or estimates) at 222 sites (49%). If past counts are representative of current population sizes, this indicates a minimum total breeding population estimate of ~ 77400 pairs. Where population sizes have at some time been known, 69% of breeding sites held ≤ 100 pairs.

**Fig. 3:**
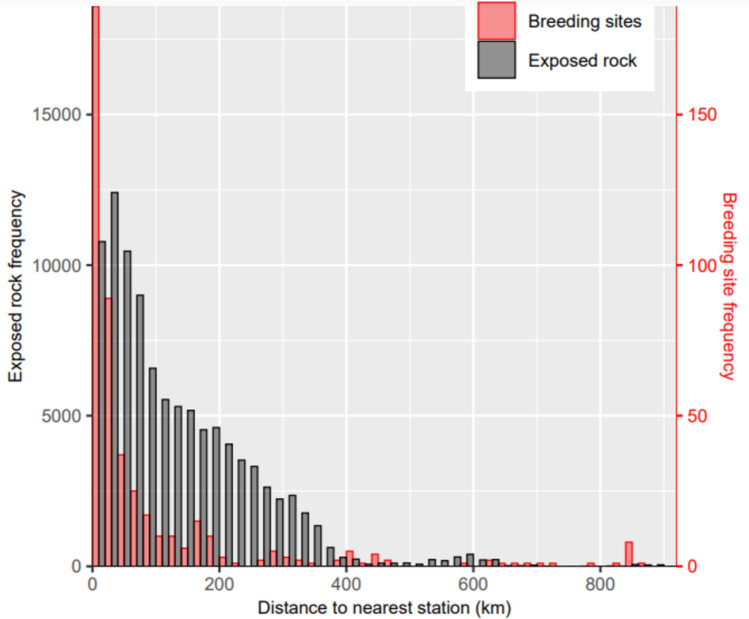
Frequency distribution of the distance between exposed rock polygons and breeding sites, and the nearest research station. Stations sourced from CONMAP 2017, Quantarctica. Exposed rock polygons sourced from Cox et al. (2023a)

Most known breeding sites are relatively close to research stations (median distance = 25.96 km, IQR = 8.53 to 81.76 km, range = 0.32 to 875.38 km; Fig. 3), with 406 breeding sites (86%) < 200 km from the nearest station, and 297 (65%) < 50 km from the nearest station. However, much exposed rock (a requirement for nesting) is available beyond 50 km from stations where considerably fewer sites are reported, and unknown breeding sites may exist.

### Local environmental conditions

**Fig. 4:**
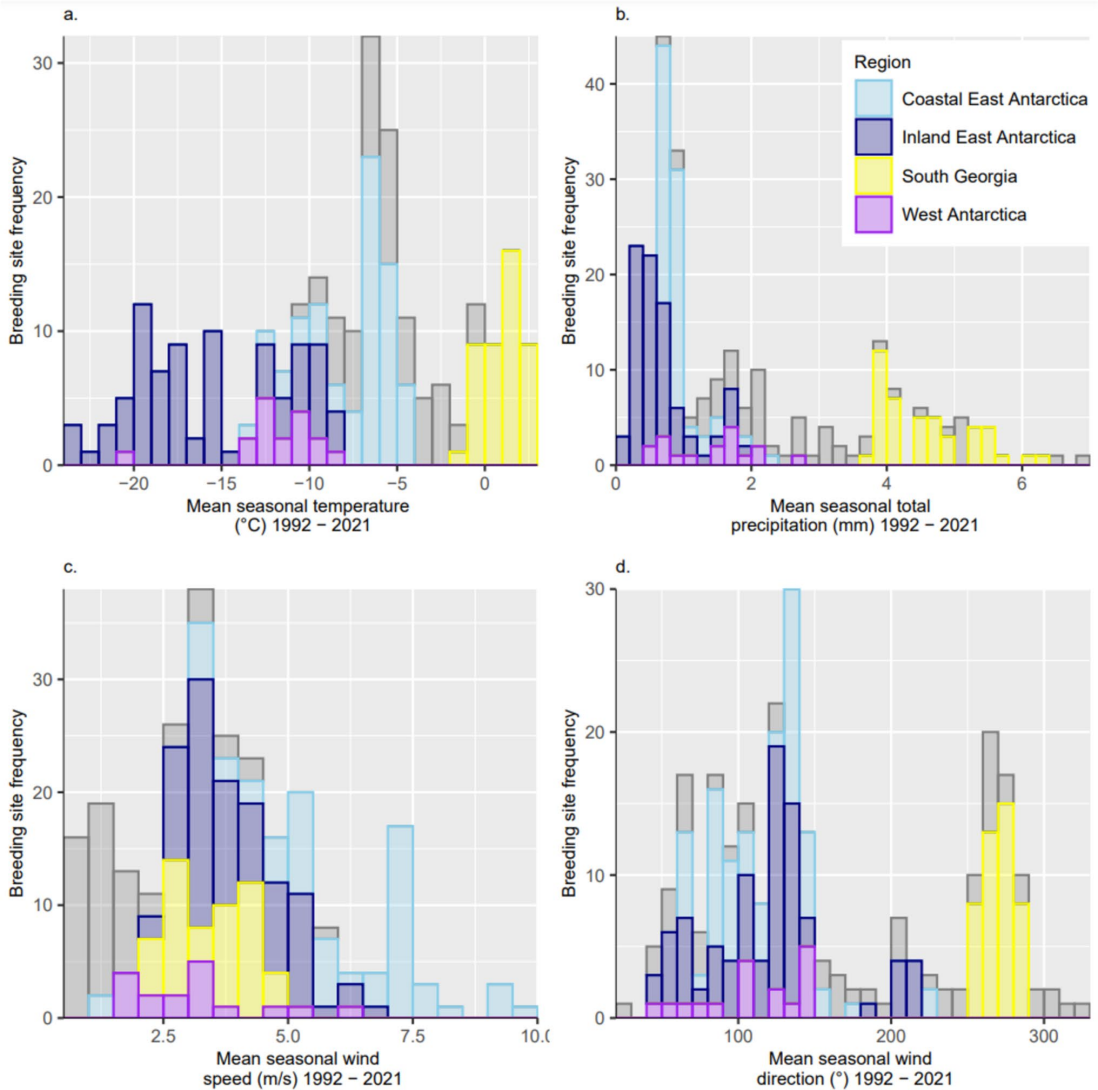
Frequency distributions of mean seasonal climate variables at snow petrel breeding sites in 1992–2021, by region. **a** 2 m surface temperature ( °C), **b** total precipitation (mm), **c** wind speed (m/s), **d** wind direction (°). Climate data sourced from Muñoz Sabater (2019), accessed January 2023

There was extensive variation in environmental conditions at breeding sites (Fig. 4, Table 1), with a median temperature of − 6.9 °C (IQR = − 12.8 to − 4.2 °C, range = − 23.8 to 2.9 °C, *n* = 247), total precipitation of 1.0 mm (IQR = 0.7 to 3.1 mm, range = 0.1 to 6.9 mm) and seasonal wind speed of 3.5 ms^−1^ (IQR = 2.5 to 4.9 ms^−1^, range = 0.5 to 10.0 ms^−1^). The mildest climatic conditions are experienced at South Georgia (the northern breeding limit), where mean seasonal temperatures and total precipitation were > 0 °C and > 3.0 mm, respectively, but mean wind speeds were similar to the median for all sites. On the Antarctic Peninsula, mean seasonal surface temperatures vary between − 10 and 0 °C, and total precipitation between 0.5 and 7.0 mm, with warmer and wetter conditions closer to the west coast. The lowest, most extreme mean seasonal temperatures are experienced at inland Antarctic breeding sites, varying between − 23.8 and − 4.0 °C, whereas mean seasonal wind speeds are highest at sites in coastal East Antarctica.

**Table 1:** Local climate variables at snow petrel breeding sites during the austral summer (November -February) in 1992–2021

	Mean	Standard deviation	Range	Median	Interquartile range
All Sites (*n* = 247)					
Mean seasonal air temperature (°C)	− 8.3	6.7	− 23.8–2.9	− 6.9	− 12.8–− 4.2
Mean seasonal total precipitation (mm)	1.9	1.7	0.1–6.9	1.0	0.7–3.1
Mean seasonal wind speed (m/s)	3.8	2.0	0.5–10.0	3.5	2.5–4.9
Antarctic Continent (*n* = 150)					
Mean seasonal air temperature (°C)	− 12.0	5.5	− 23.8–− 4.1	− 10.8	− 17.2–− 6.7
Mean seasonal total precipitation (mm)	0.8	0.5	0.1–2.6	0.8	0.50–1.0
Mean seasonal wind speed (m/s)	4.6	1.8	1.1–10.0	4.3	3.2–5.7
Antarctic Peninsula (*n* = 54)					
Mean seasonal air temperature (°C)	− 5.4	2.4	− 11.0–− 0.6	− 5.4	− 7.1–− 3.5
Mean seasonal total precipitation (mm)	2.8	1.5	0.8–6.9	2.3	1.8–3.5
Mean seasonal wind speed (m/s)	1.7	1.1	0.5–5.9	1.3	0.9–1.9
South Georgia (*n* = 43)					
Mean seasonal air temperature (°C)	1.0	1.1	− 1.5–2.9	1.1	0.2–1.7
Mean seasonal total precipitation (mm)	4.5	0.7	3.8–6.2	4.4	4.0–4.8
Mean seasonal wind speed (m/s)	3.5	0.8	2.4–4.8	3.7	2.7–4.3

**Fig. 5:**
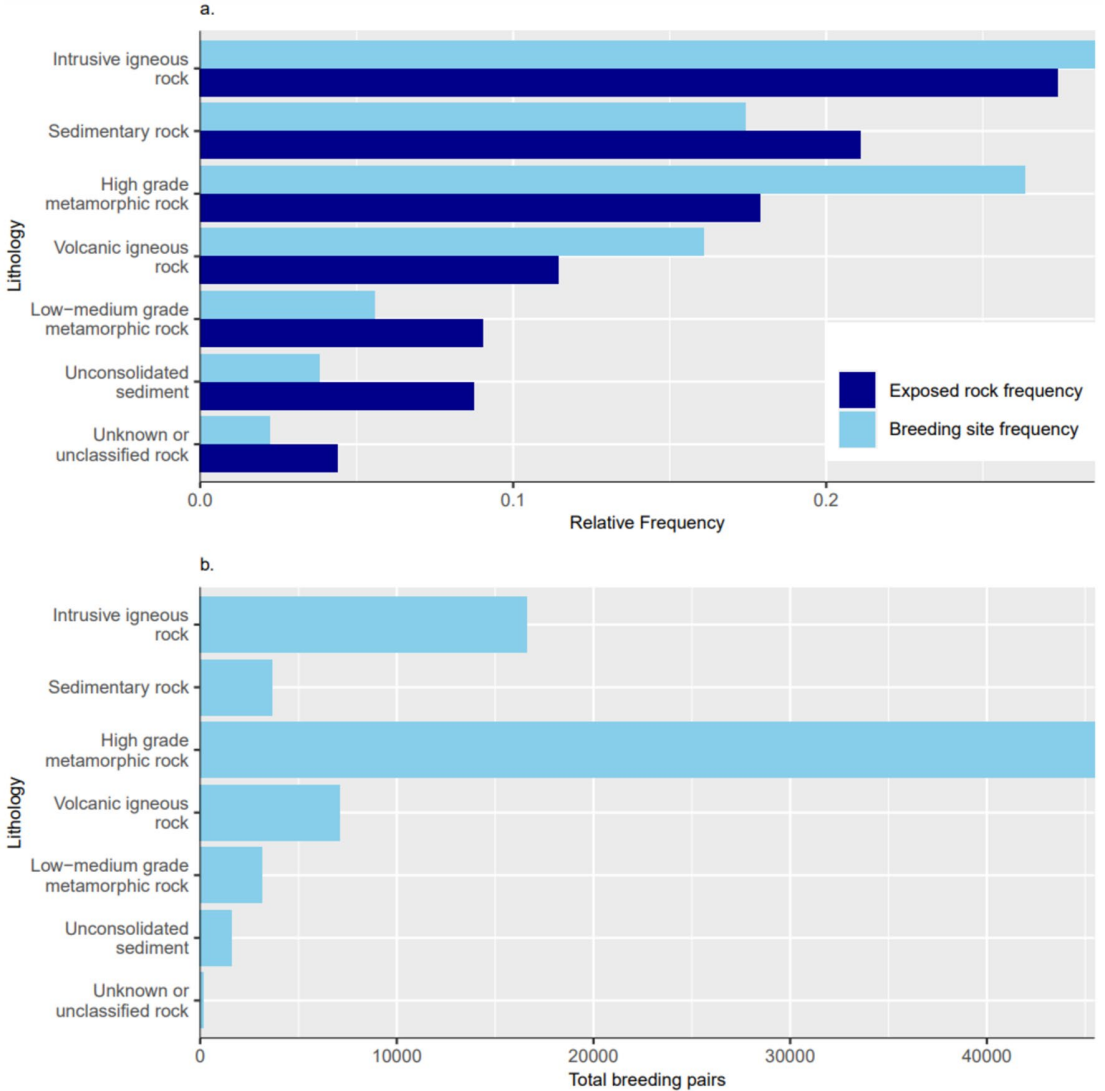
**a** Relative frequency distribution of lithology at snow petrel breeding sites, compared to the relative frequency distribution of the lithology of exposed rock polygons across the Antarctic. **b** Total number of breeding pairs of snow petrels on each lithology. Lithological data from Cox et al. (2023a)

The most available lithology by frequency in Antarctica is intrusive igneous (27%), followed by sedimentary (21%) and high-grade metamorphic rock (18%) (Fig. 5a). Breeding sites are found most often on intrusive igneous rock (28%) and high-grade metamorphic rock (26%). Fewer sites are on sedimentary rock (17%) despite its relatively high availability (Fig. 5a). For the 222 breeding sites with population estimates, the number of breeding pairs on high-grade metamorphic rock (> 45000 pairs) outnumbers the total pairs on intrusive igneous rock (< 17000 pairs) or any other lithology.

### Regional sea-ice conditions

**Fig. 6:**
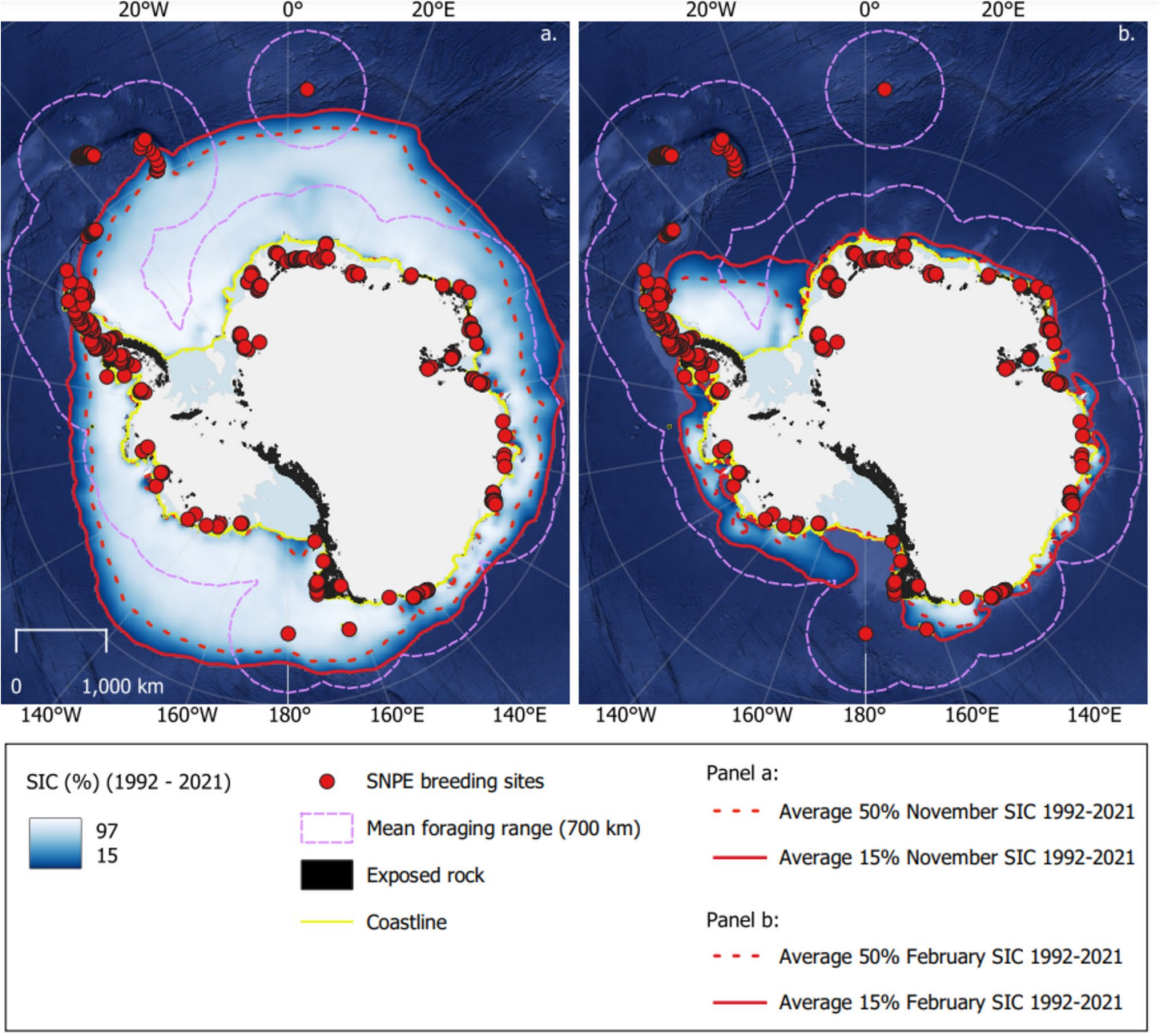
**a** Mean November sea-ice concentration [SIC] in 1992–2021 within foraging ranges of known snow petrel breeding sites. **b** Mean February SIC in 1992–2021 within foraging ranges of known snow petrel breeding sites

Sea-ice conditions in foraging areas accessible to breeding snow petrels differed between regions and during the breeding months (Fig. 6). Breeding sites on Bouvet Island, the South Shetland Islands, South Orkney Islands, South Sandwich Islands, and South Georgia, are at or beyond the 30-year average November ice edge contour (Fig. 6a). The likely foraging habitat is therefore very different to sites with accessible foraging areas within the MIZ. We have therefore quantified foraging-habitat use only for breeding sites where the birds likely feed within the MIZ (*n* = 333).

**Fig. 7:**
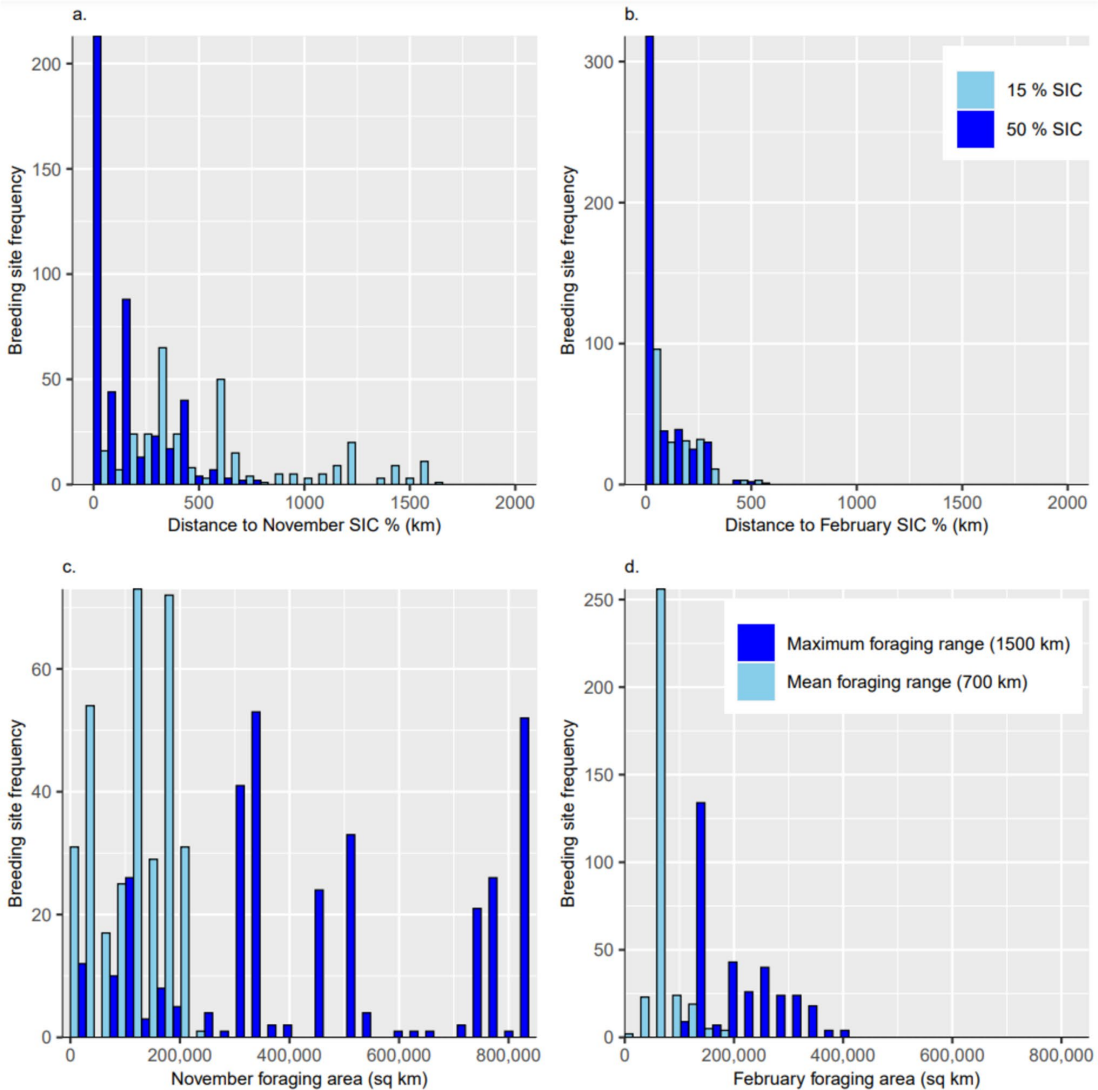
Foraging habitat of snow petrel at key months in the breeding season (November = arrival and laying; February = mid-late chick-rearing) in 1992–2021. Frequency distribution of distance from breeding sites to 15% and 50% SIC contours in **a** November, and **b** February. Frequency distribution of the extent of foraging areas based on mean and maximum foraging ranges in **c** November, and **d** February. Foraging area is calculated as the total area of sea ice between 15 and 50% concentration

In November, when SIE is at its maximum during the breeding season, the median distance from breeding sites to the ice edge is 430 km (IQR = 295 to 694 km, range = 6 to 1682 km), and to the 50% SIC contour is 136 km (IQR = 30 to 282 km, range = 1 to 737 km) (Figs. 7a, b). These are generally within the mean foraging range (~ 700 km) and well within the maximum foraging range (1500 km). The 15–50% SIC zone lies beyond the mean foraging range only for inland breeding sites in Dronning Maud Land, the Transantarctic Mountains, and Marie Byrd Land. The November 50% SIC contour only reached the coast adjacent to coastal-breeding sites east and west of Amery Ice Shelf, Adélie Land, and north of the Ross Ice Shelf (Fig. 6a). Within the assumed mean foraging range, the median area of sea ice between 15 and 50% SIC in November is 113000 km^2^ (IQR = 42400 to 167000 km^2^, range = 4,520 to 237000 km^2^). Within the maximum foraging range, the median foraging area is 396000 km^2^ (IQR = 325000 to 762000 km^2^, range = 19500 to 841000 km^2^).

Between November and February, the ice edge retreats towards the continent by hundreds of km (mean = 472 km, standard deviation = 344 km, range = − 8 to 1248 km). The greatest retreat is north of Dronning Maud Land (> 1000 km). By February, the most extensive and highest concentration remaining sea ice (> 90% SIC) is in the Weddell and Bellingshausen Seas, and adjacent to the coast of North Victoria Land; these are all areas with no or relatively few known snow petrel breeding sites (Fig. 6b). The median distance from breeding sites to the February ice edge is 47 km (IQR = 21 to 163 km, range = 0.3 to 564 km), and to the 50% SIC contour is 27 km (IQR = 10 to 136 km, range = 0.1 to 535 km) (Figs. 7c, d). Within the assumed mean foraging range, the median area of sea ice between 15 and 50% SIC in February is 60,900 km^2^ (IQR = 46700 to 67600 km^2^, range = 4,840 to 174000 km^2^), and within the maximum foraging range, the median area of 15–50% SIC is 201,000 km^2^ (IQR = 146000 to 265000 km^2^, range = 110000 to 398000 km^2^).

## Discussion

### Geographic distribution

More snow petrel breeding sites are known within East Antarctica (69 breeding sites, between 76°E and 112°E), and the north-west Antarctic Peninsula (61 breeding sites, between 61°S and 69°S) than in other regions (Fig. 1). From available population estimates, East Antarctica has held the highest numbers of breeding pairs (at least 21160), followed by the South Orkney Islands (at least 20129 pairs, including 20000 on Laurie Island) (Clarke 1906). As a loosely colonial cavity-nesting species, defining the extent of a snow petrel breeding site and colony is difficult, and many population sizes may be underestimated. However, the population estimate for Laurie Island probably represents multiple colonies (Coria et al. 2011). The count data that we collated were collected using a variety of methods, ranging from highly subjective estimates by non-specialists (e.g., Greenfield and Smellie 1992) to dedicated surveys (e.g., Johansson and Thor 2004; Olivier and Wotherspoon 2008; Pande et al. 2020). These data were collected over a period spanning 117 years, the majority between 1960 and 2020. During this period, there has been marked environmental change in some regions (e.g., Turner et al. 2016; Fogt et al. 2022; González-Herrero et al. 2022). Furthermore, longitudinal population data are available from very few sites (e.g., Chastel et al. 1993; Barbraud et al. 2000; Jenouvrier et al. 2005). For these reasons, we cannot produce a reliable estimate of the current size of the global snow petrel population.

The breeding distribution in relation to distance to the coast suggests that the furthest inland breeding site at Greenall Glacier (440 km inland) is an outlier compared with the 323 breeding sites that are ≤ 10 km from the coast. However, the distance from breeding sites to the MIZ, their main foraging habitat, is more biologically relevant. At “Skiltvakta” in the Shackleton Range (Transantarctic Mountains), breeding is unconfirmed, but this is 1680 km and 740 km from the ice edge and 50% SIC contour, respectively, in November. Therefore this site, relative to accessible foraging habitat, is more remote. In total, 64 breeding sites in the Transantarctic Mountains and Dronning Maud Land are > 1000 km from the November sea-ice edge.

### Regional absences

Our review of known breeding sites highlights that there are extensive regions of exposed bedrock where nesting has not been recorded. These gaps could be due to lack of search effort or true absences. Notably, no sites have been recorded on the eastern Antarctic Peninsula south of 69°05’S, adjacent to the western edge of the Weddell Sea (Fig. 1). This contrasts with the rest of the Antarctic Peninsula, a region of relatively high seabird abundance (Schrimpf et al. 2020), with at least 89 snow petrel breeding sites and minimum of 1264 breeding pairs. Similarly, only 8 breeding sites are known in Victoria Land, one of continental Antarctica’s biggest ice-free regions. With a large proportion of exposed low-elevation coastal bedrock (Kim et al. 2015), the number of breeding sites here is thus unlikely to be limited by bedrock availability. Furthermore, the disparity between the estimated number of breeding pairs from land-based observations in Victoria Land and adjacent islands (~ 5300 pairs; this study) and the estimate of 1.97 million snow petrels in the Ross Sea region based on densities recorded at sea (Ainley et al. 1984), seems likely to indicate there are numerous unknown breeding sites in this area.

Our results show there is a systematic decrease in the number of breeding sites in areas of bedrock with distance from the nearest research station, demonstrating a geographical bias in knowledge and survey effort that is clearly related to human presence, likely due to logistical constraints (Fig. 3). Though research stations are also located predominantly at coastal sites with exposed rock, snow petrels are confirmed to breed up to 440 km inland. Thus, the lack of breeding sites further from stations (and further inland) where bedrock remains available (Fig. 3), suggests it is highly likely that these more distant areas are under-sampled, and that many remote sites remain undiscovered. This would explain obvious gaps in the circumpolar breeding distribution in North and South Victoria Land, where exposed bedrock is readily available and at-sea density distributions suggest there are millions of snow petrels, but only 8 breeding sites are known.

From several surveys, snow petrel absence sites have been inferred with a varying degree of certainty. In East Antarctica, 5 small unnamed islands within the Davis Islands, 10 sites within the Larsemann Hills, and 6 sites within the Haswell Archipelago were surveyed and no evidence of breeding was detected (Melick et al. 1996; Pande et al. 2020; Golubev 2022). Similarly, snow petrels apparently do not breed at Jutulrora and Straumsvola in Dronning Maud Land (Ryan and Watkins 1988), nor Vesleskarvet (Steele and Hiller 1997). In Adélie Land, surveys found no evidence of breeding at 9 sites along the coast and 3 sites on inland mountains (Barbraud et al. 1999). A partial survey of Southern Masson in the Framnes Mountains (inland Prince Charles Mountains) also found no snow petrel nests (Olivier and Wotherspoon 2008). These sites with no evidence of breeding are close to regions where snow petrels do breed (e.g., 12 known breeding sites in the Larsemann Hills, summing to > 470 breeding pairs). Hence the distribution of confirmed absences is insufficient to explain any large regional gaps in Fig. 1. The proximity of presences and absences suggests that regional sea-ice conditions are likely to be the same, so that distance to suitable foraging habitat is unlikely to be a limiting factor that would explain why breeding does not take place (Ainley et al. 1984). Instead, it is possible these local absences reflect nesting-habitat availability or preferences, as follows.

### Potential environmental limits on breeding distribution

The selection of a suitable nest site is a critical decision for any bird (Stauffer and Best 1982). As central-place foragers breeding on land and foraging at sea, snow petrels face a distance-dependent cost of accessing food, and seabird populations in general are regulated by bottom-up processes and food availability (Wakefield et al. 2014; Sauser et al. 2021b). Breeding sites may therefore be chosen based on the quality and proximity of foraging habitat (Bolton et al. 2019), as well as the suitability of local nest sites (Li and Martin 1991; Lõhmus and Remm 2005). Ainley et al. (1984) hypothesised that the snow petrel breeding distribution is affected by the existence of accessible pack ice during the breeding season. Our results support this hypothesis, given the distribution of distances from breeding sites to 15% SIC and 50% SIC in November (medians of 430 and 136 km, respectively). As such, the persistence of high SIC in the western Weddell Sea, which is highly variable in extent but survives summer melt (Fig. 6b; Turner et al. 2020), could explain the lack of breeding sites on the eastern Antarctic Peninsula.

At a local scale, snow petrels are constrained to pre-existing cavities provided by the substrate (Ramos et al. 1997). They are therefore subject to intraspecific, as well as interspecific competition for these resources with other seabirds that have a similar habitat preference (Lõhmus and Remm 2005; Wiebe 2011). The availability of suitable cavities is inherently linked to rock type, jointing, and weathering. Our results demonstrate that snow petrels breed most frequently in cavities in high-grade metamorphic and intrusive igneous rocks (Fig. 5a). Estimated breeding population sizes (recent past and present) are highest on high-grade metamorphic rocks, despite the higher availability of igneous intrusive and sedimentary rocks (Fig. 5), suggesting that metamorphic rocks are more likely to incorporate suitable cavities. Additionally, specific selection of lithologies by snow petrels at a local scale is implied at multiple localities. At Edisto Inlet in Cape Hallett, no suitable cavities were observed on the eastern cliffs composed of volcanic rocks, whereas over 6 miles of the western cliffs, composed of fine-grained metamorphic rock, were occupied extensively by snow petrel nests (Maher 1962). Frequent strong winds and precipitation at this locality during the 1960/61 austral summer resulted in nesting cavities being buried by snow (Maher 1962). Therefore, it is unlikely that nests on the western cliffs were selected due to favourable aspect, but that there were no suitable cavities in the eastern volcanic cliffs. By contrast, in the northern Prince Charles Mountains, relatively few snow petrels nest in the high-grade metamorphic rock (Precambrian basement gneisses), despite it being the dominant exposed bedrock in the region. Instead, the majority of known sites are in the Amery group sandstones, where suitable cavities form through salt wedging (Heatwole et al. 1991). Furthermore, Verkulich and Hiller (1994) suggest that snow petrels in the Bunger Hills select mainly metamorphic and igneous rocks for nesting, since they are least susceptible to weathering, but also highlight the importance of aspect for protection against strong winds and snow accumulation. Therefore, we hypothesise that lithology, specifically the availability of high-grade metamorphic and intrusive igneous rocks, is an important local-scale control on snow petrel nesting-habitat selection, given its association with both cavity availability and durability.

In the predominantly high-grade metamorphic mountains of Dronning Maud Land (Cox et al. 2023a), cavity availability is unlikely to be limiting the breeding distribution. Here, observations report most breeding sites face north, which may provide shelter from katabatic winds and therefore a more favourable microclimate (Bowra et al. 1966; Mehlum et al. 1988; Ryan and Watkins 1989; Johansson and Thor 2004). Nests with a favourable aspect have higher breeding success (Olivier et al. 2005). Therefore, where the availability of cavities is not limited, interplay between nest aspect and local climate may determine nest site selection (Olivier and Wotherspoon 2006).

Based on these results, breeding location and cavity selection by snow petrels is likely to be driven by a hierarchy of regional and local environmental conditions, most importantly limited by suitable breeding substrate availability (bare rock) within a sustainable distance of suitable foraging habitat (MIZ) (Ainley et al. 1984). At locations within the foraging range of suitable foraging habitat, snow petrels may then select specific cavities based on availability (related to lithology), and local conditions such as cavity size (for predation protection) and aspect (Olivier and Wotherspoon 2006). Therefore, models of habitat selection that incorporate both distance to the MIZ and the availability of exposed high-grade metamorphic rock could be used to estimate the breeding distribution of snow petrels throughout their range.

### Past and future breeding distribution

Radiocarbon dates from snow petrel stomach-oil deposits—thick, layered accumulations outside nests—demonstrate discontinuous but persistent occupation of breeding sites throughout Dronning Maud Land, East Antarctica, the Shackleton Range and Prince Charles Mountains, since before the Last Glacial Maximum [LGM] and throughout the Holocene (Hiller et al. 1988; Thor and Low 2011; Berg et al. 2019a, b; McClymont et al. 2022). Conditions at these breeding sites and in foraging areas must have remained favourable during this period to facilitate nesting. However, the reconstructed LGM summer sea-ice edge was located beyond the modern foraging range, so it has been proposed that coastal polynyas within the sea ice, or at ice-shelf fronts, must have provided suitable foraging habitat (Thatje et al. 2008; McClymont et al. 2022). Although these ice-free areas may have supported large population sizes during the LGM (Carrea et al. 2019), such populations are hypothesised to have been reproductively isolated, resulting in the evolution of two morphologically distinct snow petrel subspecies (Jouventin and Viot 1985; Robert and Schön 2017; Carrea et al. 2019). During our review of breeding records, presence of the lesser (*P. n. nivea*) vs greater (*P. n. confusa/major*) snow petrel was rarely distinguished, so their relative breeding distributions remain poorly quantified. A summary of the distribution of most known forms is given in Hobbs (2019), though that compilation omits known lesser snow petrels breeding on Cockburn Island (Cowan 1981).

Snow petrels respond to environmental factors operating both at breeding sites and in foraging areas, and, as high-trophic-level predators, their breeding and foraging success are potentially valuable indicators of ecosystem health (Sydeman et al. 2012; González-Zevallos et al. 2013). Climate-driven changes in either breeding or foraging habitats could drive changes in the snow petrel breeding distribution. Most commonly, the effects of climate on seabirds are indirect and bottom-up, driven by spatiotemporal changes in prey distributions resulting from climate-driven changes in the pelagic environment (González-Zevallos et al. 2013). Seabird distributions in the future could be limited or expand in association with changes in prey availability or meteorological conditions at breeding sites, which are likely to be regionally specific (Gonzalez et al. 2023). Snow petrel population size is hypothesised to be negatively affected by a reduction in SIE (Jenouvrier et al. 2005). Winter sea ice is necessary to maintain Antarctic krill *Euphausia superba* and so its extent and duration affects abundance and food supply for snow petrels during the following summer (Loeb et al. 1997). Greater than average winter SIE thus improves the survival and breeding performance of snow petrels (Barbraud et al. 2000; Barbraud and Weimerskirch 2001; Jenouvrier et al. 2005). Summer SIE also affects their breeding success, which is depressed if November SIE is lower, whilst fledgling body condition is higher when the November SIE is greater than average (Barbraud and Weimerskirch 2001). Despite the surprising stability overall of Antarctic SIE over the past decades, there have been major declines and record minima in both winter and summer SIE in recent years, and the trend of more extreme lows is predicted to continue (Fogt et al. 2022; Raphael and Handcock 2022). Dependence of snow petrels on the proximity of the MIZ suggests that with the projected southwards retreat of SIE, they will lose substantial areas of foraging habitat. The small snow petrel population size at their northern limit on South Georgia (~ 3000 breeding pairs) is suggested to result from limited sea ice nearby during the breeding season (Ainley et al. 1984). Regional variability in future sea-ice trends (Purich and Doddridge 2023) may result in abandonment of breeding sites in some regions as foraging habitat becomes unsuitable, resulting in a southwards contraction of the breeding distribution.

In contrast, new exposed coastal-breeding habitats may emerge as the climate warms. A high proportion (71%) of known snow petrel breeding sites are ≤ 10 km from the coast. As such, increased availability of ice-free rock may increase the options for snow petrels to expand in these areas, although they may also face competition for this habitat from other seabirds.

Direct climate effects (extreme weather events) can also impact seabird distributions and breeding success at a local scale. Nesting cavities shelter snow petrels to some extent from extreme weather, but the timing and duration of local snow accumulation nevertheless influences breeding success (Croxall et al. 2002; Einoder et al. 2014), breeding probability (Chastel et al. 1993), hatching success (Olivier et al. 2005), and fledging probability (Sauser et al. 2021b). Increased or prolonged snowfall can affect nest accessibility, and a simultaneous increase in local temperatures increases the risk of flooding (Chastel et al. 1993). Extreme storm activity (severe winds and high precipitation) in Dronning Maud Land during the 2021/22 austral summer caused near-complete breeding failure and mass mortality of snow petrels and conspecifics across multiple breeding sites extending over > 700 km (Descamps et al. 2023). Mass mortality events can have major lasting effects on long-lived seabirds which are slow to reproduce (Mitchell et al. 2020), with the distributions of some (e.g., black-legged kittiwakes *Rissa tridactyla*) known to change as a result of poor breeding performance in particular areas (Boulinier et al. 2008). However, the only long-term demographic studies of snow petrels are at the Pointe Géologie Archipelago (Adélie Land) and Reeve Hill, Casey Station (East Antarctica) (Fig. 1). Most long-term studies conclude intraspecific differences between sexes and neighbouring breeding sites in responses to local weather effects and larger scale climatic patterns (Sauser et al. 2021a). Therefore, longer-term impacts of extreme breeding season weather, such as intensive storms, on the snow petrel breeding distribution remain uncertain. By quantifying average climatic conditions at breeding sites, we provide important baseline data against which future distributional shifts can be assessed. Our study highlights the need for much more widespread long-term monitoring of snow petrel colonies, including at least population trends and breeding success, and ideally, long-term demographic studies. In addition, tracking studies and the development of species distribution models of habitat suitability in foraging areas would help in predicting the future distribution of snow petrels in relation to climate-driven change.

## Data Availability

Our data set is held with UK Polar Data Centre (Francis et al. 2024) and is available at https://doi.org/10.5285/3155805f-6d8b-4a27-8dbf-91f2c10a4ba7. We hope it will facilitate ongoing research and conservation.
